# Index of Diseases and Their Treatment

**Published:** 1877-12

**Authors:** John Hawkes Tanner


### INDEX OF DISEASES AND THEIR TREATMENT.

BY JOHN HAWKES TANNER, M. D., F. L. S.

A Second edition of this useful work, revised by W. H. Broadbent, M.D. has been published by Lindsey & Blakiston, Philadelphia. It was originally prepared for the purpose of affording the practitioner whose visiting list consumed so much of his attention as to prevent him from satisfactorily consulting voluminous text-books, a convenient and reliable reference. All of the known diseases are arranged alphabetically, after the manner of an enclycopædia, with brief description of their symptoms, and directions for their treatment, which are full enough to refresh the memory of any educated physician, and bring to his mind all the knowledge he ever possessed concerning them. Under the head of treatment, the reader is referred to the proper medication and dose in the appendix of formulæ, containing several hundred prescriptions, classified according to their physiological actions. The appendix furthermore contains much valuable information aside from the formulæ; being prefaced by important suggestions in regard to the administration of medicines; also a scale for determining the appropriate dose according to age, sex, etc., besides rules to be observed during the course of disease.

Another section treats on electro-thereapeutics, wherein is given the different forms of eclectricity used in medicine, modes of application, and the diseases to which they may be applied.

The concluding portions of the book furnish information pertaining to climates for invalids. Besides some general observations upon the subject, it refers to those most favorable to different diseases, giving the peculiarieties of temperature, atmosphere, etc., with a description of the physical aspects of each. This work, strictly confined to the objects for which it is intended, is an excellent one, and well worthy of the attention of the general and special physician.

